# Analysis of fractal electrodes for efficient neural stimulation

**DOI:** 10.3389/fneng.2013.00003

**Published:** 2013-07-12

**Authors:** Laleh Golestanirad, Behzad Elahi, Alberto Molina, Juan R. Mosig, Claudio Pollo, Robert Chen, Simon J. Graham

**Affiliations:** ^1^Department of Physical Sciences, Sunnybrook Research InstituteToronto, Canada; ^2^Department of Medical Biophysics, University of TorontoToronto, Canada; ^3^Laboratory of Electromagnetics and Acoustics, Electrical Engineering, Ecole Polytechnique Fédérale de LausanneLausanne, Switzerland; ^4^Department of Neurology, Toronto Western Research Institute, University of TorontoToronto, Canada; ^5^Department of Neurosurgery, University Hospital of BernBern, Switzerland

**Keywords:** neural stimulation, fractal geometry, electrodes, epidural spinal cord stimulation, cortical stimulation, deep brain stimulation (DBS)

## Abstract

Planar electrodes are increasingly used in therapeutic neural stimulation techniques such as functional electrical stimulation, epidural spinal cord stimulation (ESCS), and cortical stimulation. Recently, optimized electrode geometries have been shown to increase the efficiency of neural stimulation by increasing the variation of current density on the electrode surface. In the present work, a new family of modified fractal electrode geometries is developed to enhance the efficiency of neural stimulation. It is shown that a promising approach in increasing the neural activation function is to increase the “edginess” of the electrode surface, a concept that is explained and quantified by fractal mathematics. Rigorous finite element simulations were performed to compute electric potential produced by proposed modified fractal geometries. The activation of 256 model axons positioned around the electrodes was then quantified, showing that modified fractal geometries required a 22% less input power while maintaining the same level of neural activation. Preliminary *in vivo* experiments investigating muscle evoked potentials due to median nerve stimulation showed encouraging results, supporting the feasibility of increasing neural stimulation efficiency using modified fractal geometries.

## Introduction

Electrical stimulation of the nervous system is a technique used to restore function to individuals with neurological impairment. Implanted planar electrodes have been used extensively in the past few years for efficient stimulation of both central and peripheral nervous systems, in many applications. Epidural spinal cord stimulation (ESCS), for example, uses electrical stimulation of the dorsal roots and/or the dorsal columns of the spinal cord to address various pain syndromes (Logé et al., 2011). Recently, the combination of ESCS and partial weight bearing therapy has been shown to induce functional gains in over-ground gait for individuals with chronic, incomplete spinal cord injury who have very low motor scores in their lower limbs (Huang et al., 2006). Within the brain, epidural cortical stimulation (ECS) provides useful intervention for neuropathic pain (Rasche et al., 2006), movement disorders (Kleiner-Fisman et al., 2003), Parkinson's disease (Canavero et al., 2002), and stroke rehabilitation (De Kroon et al., 2002; Levy et al., 2008). Deep brain stimulation (DBS), which involves high frequency electrical stimulation of the thalamic or basal ganglia structures to treat movement disorders, has rapidly emerged as an alternative to surgically created lesions (McIntyre et al., 2004). Another new, rapidly growing application is transcranial direct current stimulation (tDCS), a non-invasive, painless, safe and portable technique that modulates cortical excitability (Nitsche and Paulus, 2000). Implementation of tDCS is simple and economic: a weak DC current (<2 mA) is injected between surface electrodes that are connected to a stimulation device. The tDCS method has shown promising results as a potential therapy in stroke (Fregni et al., 2005), Parkinson's disease (Lomarev, 1996), depression (Fregni et al., 2006), and epilepsy (Liebetanz et al., 2006). Stimulation devices also have very promising applications in control of bladder function, restoring continence and micturition in some neurological diseases (Grill, 2009).

In such applications, electrodes are powered by implanted pulse generators (IPGs) which use primary cell batteries and require surgical replacement when the battery is depleted. Surgical replacement is expensive and carries substantial risk. For example, the complication rate is three times higher for replacement of cardiac pacemakers than for original device placement (Deharo and Djiane, 2005), and the complication rate for replacement of implanted defibrillators is 8.1% (Gould and Krahn, 2006).

Although much effort has been put into finding optimal anatomical targets for different nerve stimulation techniques, very little work has been done to improve the efficiency of nerve stimulation by using intelligent designs and configurations of the electrodes themselves. One attempt focused on new materials for reducing nerve stimulation thresholds, using steroid-eluting electrodes to supress inflammation and thus enhance the electrical contact at the tissue-electrode interface (Mond and Stokes, 1996; Mono and Stokes, 2006; Petersen et al., 2004). However, these electrodes retained simple square or circular shaped conductor geometry, used in single or in array configurations. It has been shown that the design of electrode geometry itself plays a significant role in controlling the activation of populations of neurons (Wei and Grill, 2009). In particular, electrode geometry can affect the spatial distribution of the electric field in tissue, and thus the pattern of neural excitation. The electrode design also impacts the effective impedance, and thus the power consumption. Reduced power requirements can extend the lifetime of existing IPGs, thus reducing both cost and risk associated with repeated IPG replacement surgeries. Alternatively, reduced power requirements could enable the use of smaller batteries, allowing IPGs to be reduced in size.

In the present work, a new family of fractal electrodes is introduced for efficient neural stimulation. Electromagnetic modeling results indicate that the proposed geometries produce a significantly higher neural activation function, providing as much as a 22% reduction in power consumption while maintaining the same percentage of neural activations achieved by conventional electrodes. Preliminary *in vivo* experiments investigating muscle evoked potentials due to median nerve stimulation showed trends that are consistent with the simulation findings.

The proposed electrode designs are straightforward to manufacture and do not require the exhaustive biocompatibility testing that is necessary when new materials are adopted. However, there is no barrier in principle to combining the fractal electrode approach with new materials, to increase the efficiency of stimulation even further.

## Materials and methods

### Overview

One approach to increase the efficiency of neural excitation by altering the electrode geometry is to maximize the neural activation function, which is proportional to the second spatial derivative of the extracellular potential, *V*_*e*_ (Rattay, 1989). The activation function *f* can be written in terms of the electric field *E* and electric current density *J* as:

(1) $$f\propto \frac{\partial^{2}V_{e}}{\partial z^{2}}=-\frac{\partial \left(E_{z}\right)}{\partial z}=-\frac{1}{\sigma }\frac{\partial \left(J_{z}\right)}{\partial z}$$

where *z* is the direction along the axon and σ is the conductivity of the medium. Because *f* is proportional to the spatial derivative of the electric field, it can be maximized by increasing the irregularity of current profile on the surface of the electrode.

Recently, planar geometries have been proposed to maximize *f* by increasing the perimeter of the electrode (Wei and Grill, 2009). An interesting alternative approach is to increase the *irregularity* of the surface current profile which can be quantified by defining a metric known as *topological edginess*. These concepts naturally lead to the development of a series of modified fractal shapes as candidate electrode geometries, which increase surface current irregularity while maintaining the amount of total current delivered to the tissue. The performance of this new family of electrodes is validated by performing a rigorous finite element analysis of field distributions in conjunction with simulations of a large neuron population to investigate the actual percentage of activation, and preliminary *in vivo* experiments.

The subsequent outline of this paper is as follows: Sections Background and Sierpinski Carpet and Concept of Capacity Dimension provide an overview of fractal geometries and the mathematical definition of *capacity dimension*, concepts that are useful in the design of high efficiency neural stimulators. Section Modified Sierpinski Carpet describes the construction process to build modified fractal electrodes, whereas Sections Finite Element Modeling and Neural Activation Prediction give the details of finite element modeling as well as neuron modeling to predict the efficiency of activation. Section Numerical Results presents the result of finite element simulations and neural modeling. Section Prototyping and *in vivo* Experiments describes preliminary *in vivo* experiments. Finally, the ramifications of the results are discussed in Section Discussion and Conclusion.

### Background

In essence, a fractal is a fragmented geometric shape that can be split into parts, with each part appearing (at least approximately) as a reduced-size copy of the whole—a property called self-similarity. The basic ideas of fractals date back to the 17th century, however rigorous mathematical treatment was introduced almost a century later by Karl Weierstrass, Georg Cantor, and Felix Hausdorff in studying functions that were continuous but not differentiable (Edgar, 2004). The term fractal itself was coined by Benoît Mandelbrot in 1975, derived from the Latin *“fractus”* meaning “broken” or “fractured” (Mandelbrot, 1983). The original inspiration for developing fractal geometries came largely from in-depth scrutiny of galaxies, cloud boundaries, snowflakes, trees, leaves, and other self-similar patterns found in nature. Applications of fractals are now widespread in many branches of science and engineering (Werner and Ganguly, 2003). For example, in fractal electrodynamics, fractal geometry has been combined with electromagnetic theory specifically to propose improved designs to control the radiation pattern, wave propagation, and scattering characteristics of radio-frequency devices (Jaggard, 1991, 1995) and a number of patents have been filed using fractal shapes to improve design of antennas or frequency selective surfaces (Cohen, 2000a,b; Uei-Ming, 2008).

At present, however, use of fractal geometries to control the static electric field distribution in tissue, desirable for improving neural stimulation, is a novel concept that remains to be explored.

### Sierpinski carpet and concept of capacity dimension

**Figure 1:**
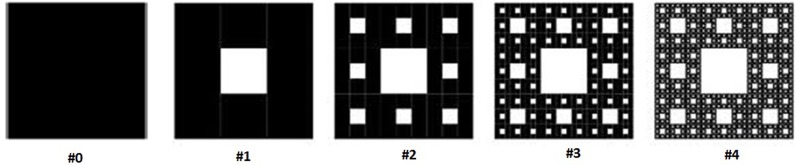
**Stages in constructing Sierpinski carpet fractals**.

This section describes mathematical properties of fractals in more detail, in the context of a specific fractal shape known as the Sierpinski carpet (Peitgen et al., 2004). The first few stages in the construction of the Sierpinski carpet are demonstrated in Figure 1. The procedure starts with a base square (stage #0). Next, the square is divided into nine congruent sub-squares in a 3 × 3 grid, and the central sub-square is removed (second stage). The same procedure is then applied recursively to the remaining eight sub-squares (third stage), and then can be repeated an infinite number of times.

One metric used to quantify the complexity of fractal shapes is called the *fractal dimension* (Falconer, 2003), given by the ratio of the change in detail to the change in scale. For sets describing ordinary geometric shapes, the fractal dimension equals the Euclidean or topological dimension: 0 for sets describing points; 1 for sets describing lines; 2 for sets describing surfaces; and 3 for sets describing three dimensional geometries. However, unlike topological dimensions, the fractal index can take non-integer values, indicating that a fractal set fills space in a quantitatively different fashion than an ordinary geometrical set (Vicsek, 1992).

In Figure 1, let *N*_*n*_ be the number of black boxes, *L*_*n*_ the length of a side of a white box, and *A*_*n*_the fractional area of black boxes after the *n*^th^ stage of construction. Assuming that the base square has a unit area equal to 1, then for *n* ≥ 0:

(2a) $$N_{n}=8^{n}$$

(2b) $$L_{n}=3^{-n}$$

(2c) $${\text{A}}_{n}=L_{n}^{2}N_{n}={\left(\frac{8}{9}\right)}^{n}$$

The capacity dimension is defined as (Mandelbrot, 1983):

(3) $$d_{capacity}=-\underset{n\to \infty }{\lim}\frac{\ln N_{n}\;}{\ln L_{n}\;}\;=1.892789$$

Notably, the capacity dimension of this fractal is less than 2, the value for a solid surface. In other words, the “topological edginess” of the geometry has been increased which, when fabricated as an electrode, is hypothesized to lead to a substantially increased activation function.

### Modified sierpinski carpet

In progression toward higher order fractals, the total metallic surface area of the electrode will inevitably be reduced. This could potentially have negative impact on the efficiency of neural excitation, as the total amount of current delivered to the tissue could be reduced. In anticipation of this problem, modified electrode geometries are considered that include systematic re-attachment of cut-out parts to ensure constant total metallic surface area, while maintaining increased surface irregularity.

**Figure 2:**
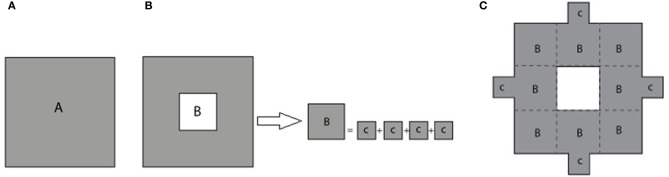
**(A)** Base square with surface area A. **(B)** The removed central square of area B is divided equally into four smaller squares of area C. **(C)** The smaller squares are attached to the periphery of the first stage standard fractal to build the first stage *modified* Sierpinski square.

**Figure 3:**
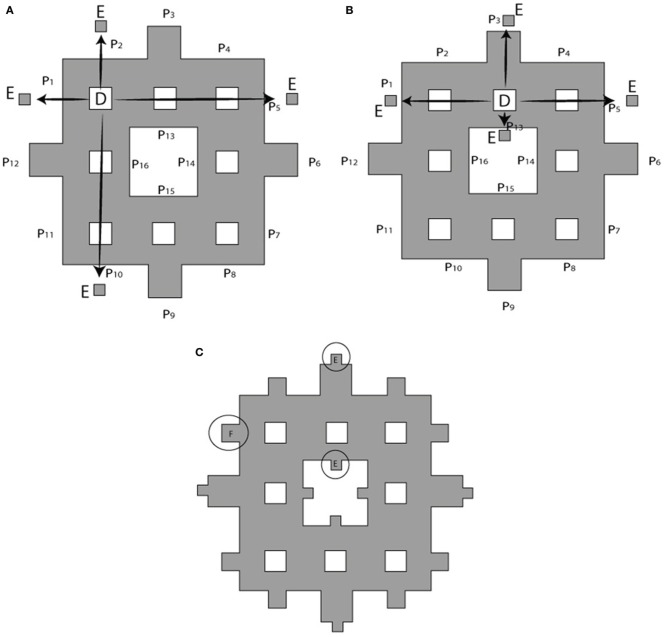
**Building a second order modified Sierpinski square. (A)** Squares of area D at the corners of a rectilinear interior pattern are cut out and divided into four smaller squares of area E = D/4 to be attached to outer boundary of electrode as shown by arrows. Target locations P1 through P15 are indicated. **(B)** Cut-outs for the remaining interior squares are distributed according to a different pattern. **(C)** Squares placed at the same location are merged into a single larger square of equivalent surface area *F* = 3 × *E*. See text for further details.

The process of constructing modified fractals is outlined below for the example of the Sierpinski carpet fractal, but also is applicable to fractal geometries such as the Sierpinski triangular gasket, Sierpinski pentagon, and others. The modification procedure starts with the base square of a surface area A (Figure 2A). Its central square of area B = A/9 is removed and divided into four equal squares (see Figure 2B). These parts are reattached to the outer boundary of the first order fractal to build the first order *modified* Sierpinski fractal (see Figure 2C). The steps to build the second order modified Sierpinski square are basically similar. Progressing toward higher order shapes, however, there are degrees of freedom regarding where to re-attach the cut-out parts at each step. One scenario is depicted here for the second order fractal, as illustrated in Figures 3A–C. The procedure starts by cutting out an additional rectilinear pattern of interior squares, each with area of D = B/9, from all sub-squares labeled B in Figure 2C. Each of these squares are then divided into four smaller squares (labeled as E) with a surface area of E = D/4. E squares from the four corner D squares are attached to positions (P_1_, P_2_, P_5_, P_10_), (P_1_, P_4_, P_5_, P_8_), (P_4_, P_7_, P_8_, P_11_), and (P_2_, P_7_, P_10_, P_11_), as shown in Figure 3A. E squares made from four middle D squares are attached to positions (P_1_, P_3_, P_5_, P_13_), (P_4_, P_6_, P_8_, P_14_), (P_15_, P_7_, P_9_, P_11_), and (P_16_, P_10_, P_12_, P_2_), as shown in Figure 3B. From these operations, three squares of surface E should be placed in each of P_1_, P_2_, P_4_, P_5_, P_7_, P_8_, P_10_, and P_11_ positions. These squares are merged into a single square of equivalent area F = 3 × E that is placed at each of the above mentioned positions, as shown in Figure 3C.

### Finite element modeling

**Figure 4:**
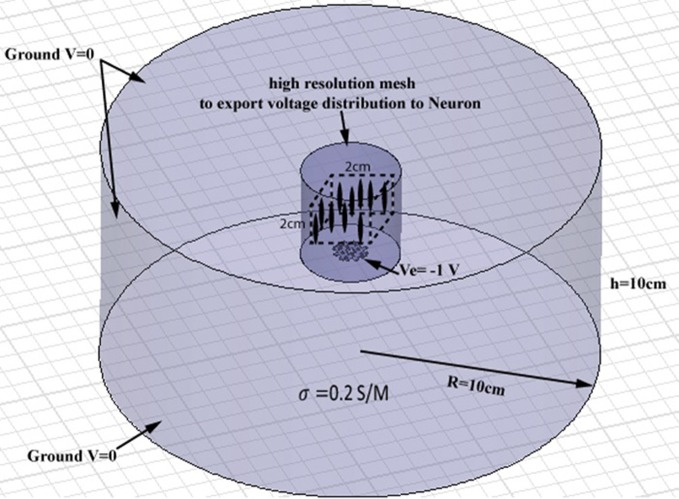
**Geometry of the finite element model of a planar electrode adjacent to a cylindrical homogenous volume conductor**. The tissue medium was modeled as a cylinder 10 cm in diameter and 10 cm in height with a conductivity of 0.2 S/m. A high resolution cylindrical region was introduced around the electrode (diameter of 40 mm and height of 40 mm) to ensure the high accuracy of FEM results needed for further simulation of neural activation. A population of 256 axons were distributed in a volume of 2 cm × 2 cm × 2 cm, placed 15 mm above the electrode (the cubic area and axons are shown schematically, not to scale).

Full-wave methods have been extensively used to address the interaction of electromagnetic waves and living tissues (Golestani-Rad et al., 2007; Golestanirad et al., 2010, 2012a,b). To investigate the performance of the proposed modified fractal electrodes, three-dimensional (3D) finite element models of fractal electrodes were developed inside a 3D homogenous conducting medium (Figure 4). The model included the electrode surface with a potential of −1 V and a homogenous volume conductor representing the neural tissue [σ = 0.2 S/m (Ranck, 1963)]. The tissue adjacent to the electrode was modeled as a cylinder with a radius of 10 cm and a height of 10 cm and the outer boundaries set to *V* = 0 to simulate the cathodic monopolar stimulation. The 3D models were implemented in Ansys Maxwell 3D and were partitioned into ≥1800000 tetrahedral elements. To ensure the high accuracy of FEM results needed for further simulation of neural activation, a high resolution cylindrical region was introduced around the electrode (diameter of 40 mm and height of 40 mm) as depicted in Figure 4 where the mesh size was <0.5 mm. The FEM solver was set to follow an adaptive iterative process whereby an initial mesh was seeded according to the geometrical details of the structure. The Maxwell3D DC conduction solver which included insulator fields computed and stored the value of the electric potentials at the vertices and midpoints of the edges of each tetrahedron in the finite element mesh. The scalar potential field *V* was calculated under the quasi-static assumption by solving Laplace equation ∇ · (ó∇ *V*) = 0 as well as the electric field according to E = −∇ *V*. After E was calculated, Maxwell3D provided solution files and an error analysis. In adaptive analysis, the solver refined the tetrahedra with the highest error, and continued solving until the stopping criterion was met. The adaptive solver refined the mesh by 30% at each iteration and iterated until the difference between two successive solutions was <0.5%.

### Neural activation prediction

The NEURON environment tool (Hines and Carnevale, 2001) was used to model a population of 256 axons distributed in a volume of 2 cm ×2 cm ×2 cm above the electrode. Neurons were modeled as 57 μm-diameter myelinated axons made of 21 nodes of Ranvier separated by 20 internodes as in other modeling studies of neural stimulation in the literature (Butson and McIntyre, 2005; Butson et al., 2006). The potential distribution *V*_0_ was extracted from the FEM model from a high-resolution cubic area located 15 mm above the surface of the electrode (Figure 4) and applied as the extracellular potential to the electrical model of the axons. A 15 mm gap was introduced to account for the presence of the peri-electrode space—a region filled with extracellular fluid that is formed in the acute phase after electrode implantation (Yousif and Liu, 2009).

A square electrode, first and second order Sieprinski carpet electrodes, and first and second order modified Sierpinski electrodes were modeled and simulated. The percentage of axons activated was computed for each simulation scenario. A time varying field potential was created by convoluting the obtained potentials by a normalized time varying electric pulse of 60 μs width, as is typically used in stimulation techniques such as DBS.

## Numerical results

### Current distribution and activation function

**Figure 5:**
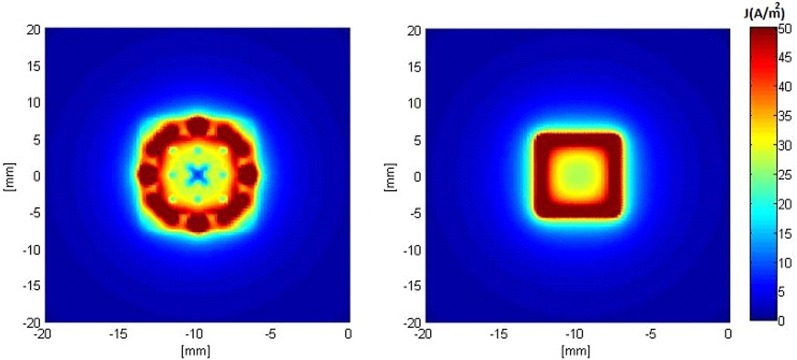
**Distributions of current density 0.5 mm above the surface of the electrodes when a constant voltage of −1 V was applied. Left:** modified second order fractal electrode, **Right:** square electrode.

**Figure 6:**
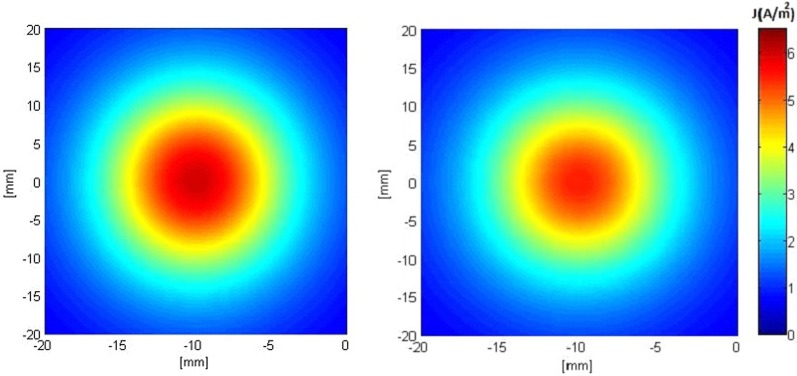
**Distributions of current density 10 mm above the surface of the electrodes when a constant voltage of −1 V was applied. Left:** modified second order fractal electrode, **Right:** square electrode.

Figures 5, 6 show the current density distribution at distances of 0.5 and 10 mm above the plane of a second order modified electrode and a conventional square electrode. At the closer distance (Figure 5) the increased irregularity of current density over the surface of the fractal electrode is readily apparent compared to that of the square electrode. The effect of fractalization is still noticeable visually at the further distance (Figure 6).

**Figure 7:**
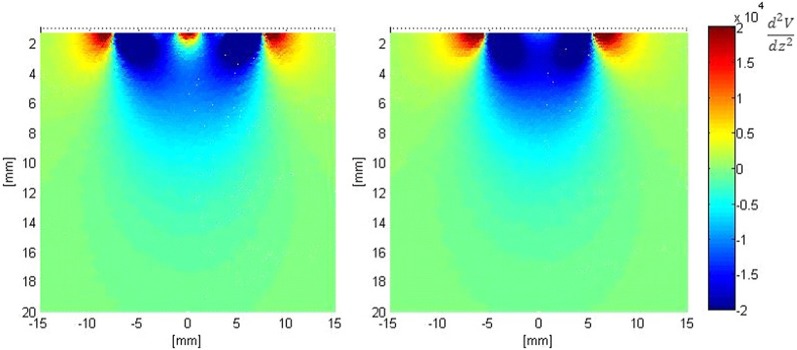
**Distribution of the second derivative of voltage along the *z*-axis in the tissue adjacent to the electrode. Left:** modified second order fractal electrode, **Right:** square electrode.

Figure 7 shows the second derivative of the electric potential V along the axis perpendicular to the surface of both electrodes (*z*-axis). From Equation 1, this quantity is proportional to the neural activation function, with positive values denoting “depolarization” and negative values denoting “hyperpolarization “of axons. These plots show that the activation function is improved in the vicinity of the fractal electrodes. Results for the first order modified electrode were similar to, but less than those for the fractal electrode presented in Figure 7 and are not shown for clarity and brevity.

### Input-output curves of neural activation

**Figure 8:**
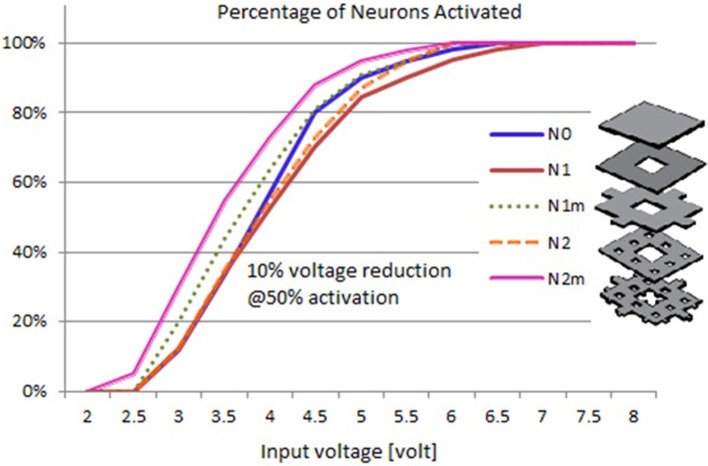
**Input–output curves of activation of model axons: percent of activated axons as a function of stimulus voltage**.

**Figure 9:**
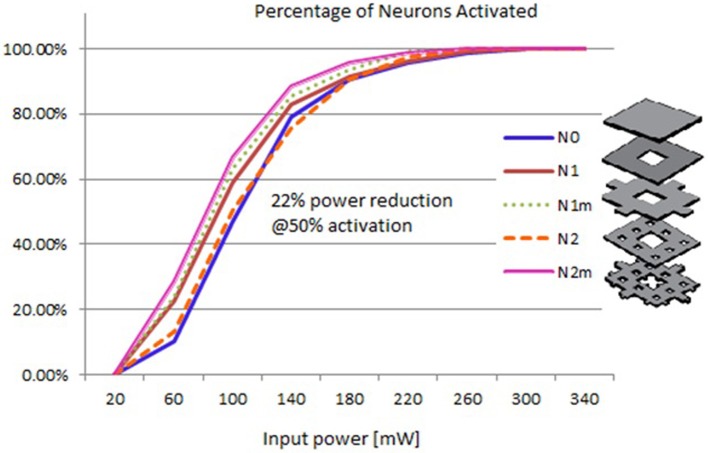
**Input–output curves of activation of model axons: percent of activated axons as a function of input power**.

**Figure 10:**
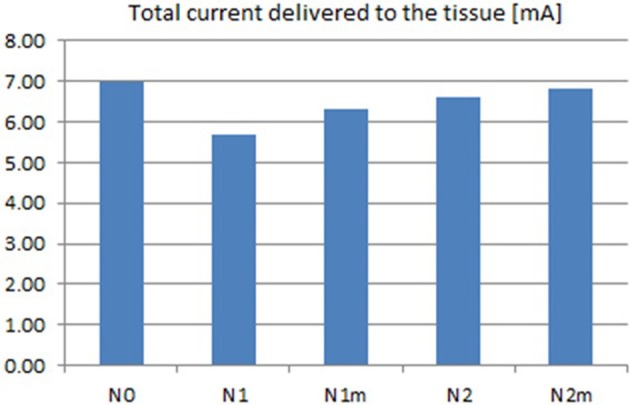
**Total current delivered to the tissue for different electrodes calculated at −1 V input excitation**. See Figure 8 for legend definitions.

Based on the modeled population of axons, as described above (also see Figure 4) input–output curves were generated of the percentage of activated axons as a function of stimulus amplitude (*V* = 2 to *V* = 8 with steps of 0.5 volt) (Figure 8) and stimulus power (Figure 9). Figure 10 gives the total current delivered to the tissue for different electrode geometries excited at −1 V input voltage. The current is calculated by taking the integral of outgoing current over the surface of the high resolution cylinder surrounding the electrode (see Figure 4). It's important to note that the total current delivered to the tissue is the result of a delicate interplay between local maxima in the current distribution and the overall metallic surface of the electrode. As a general trend, unmodified electrodes deliver less current to the tissue in comparison to modified geometries, and comparatively less activation for a fixed input voltage. However, as the total delivered current is reduced, this activation is obtained at a relatively less input power.

It can be observed that modified fractal electrode #2 decreased the average threshold voltage by 10% and decreased the average power consumption by 22%, as compared to the conventional square electrode.

## Prototyping and *in vivo* experiments

**Figure 11:**
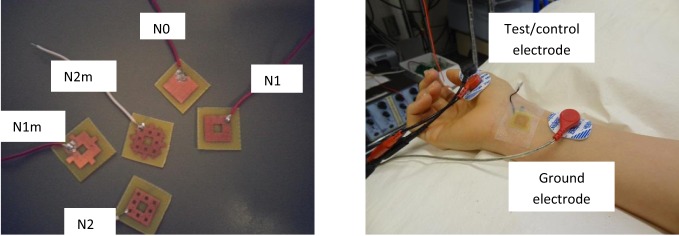
**Left:** Directly etched prototypes. See Figure 8 for legend definitions. **Right:** Experimental setup for median nerve stimulation.

To perform preliminary *in vivo* tests, above-mentioned fractal electrode patterns were fabricated in the laboratory, using a “direct etch” technique^1^. The patterns were transferred to a 1-sided, 4 mm (1/64″) thick printed circuit board (PCB) with FR-4 substrate and a 1 oz copper layer. The electrodes were etched, cut into small PCB wafers and soldered with wires for use in electrical stimulation (Figure 11). Some minor etching defects were observed in the prototypes; this problem can be easily eliminated using a professional PCB facility.

The subject was tested for muscle activities evoked by stimulation with different electrodes. Subject was blinded to the test electrodes. The protocol was approved by the University Health Network Research Ethics Board. Eectromyograms (EMGs) were recorded from the left abductor pollicis brevis (APB) muscle using bipolar electrode configuration. The signals were amplified 1000 times, filtered (5–500 Hz), digitized (Cambridge Electronic Design Micro 1401), and recorded using Signal software (version 3.07). The EMG signal was continuously monitored with visual and auditory feedback to ensure complete muscle relaxation. The median nerve was stimulated through bipolar electrodes at the wrist (cathode proximal) using a 200-μs square wave pulse. The test/control electrode was placed on the distal (anodal) part of the wrist over the median nerve. Perceptual thresholds (PTs) were measured for each test/control electrode. I/O curve were measured by recording the compound muscle action potentials from median nerve stimulation at two times of PT. Ten compound muscle action potentials were recorded and averaged for each electrode, and the amplitudes were measured peak-to-peak. For a more detailed description of recording methods used in this study the reader is referred to the section “Recording” in the chapter “Anatomy and Neurophysiology” of (Giacomini and Giacomini, 2006).

**Figure 12:**
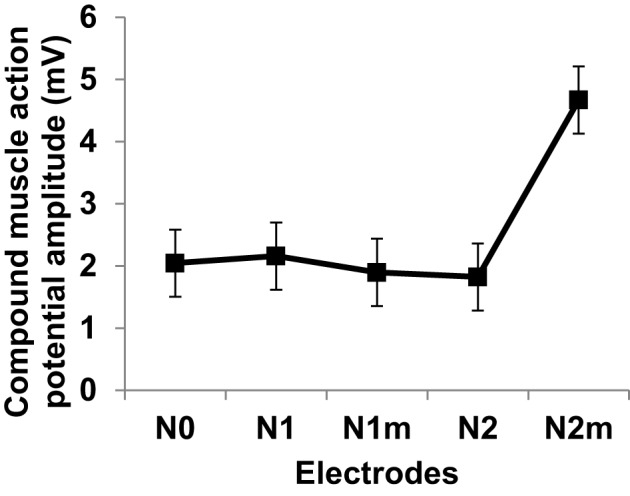
**Compound muscle action potentials amplitudes at two times perceptual threshold of median nerve stimulation**. Error bars represent standard deviation. Electrode types are as defined in Figure 8.

Figure 12 gives the average and standard deviations of the amplitudes of compound muscle action potentials recorded from stimulation using each electrode. Although the experiment setup did not closely mimic the simulation scenario of nerve stimulation, a positive trend in increasing muscle activity was confirmed for the second order modified electrode which was consistent with simulated input-output curves of Figure 8. It is important to emphasize that Figure 12 reflects the experimentation on a single subject. It is possible that slight differences between different fractal electrode types (predicted by simulations and depicted in Figure 8), can be captured only when larger groups of subjects are recruited and analysed. More comprehensive studies should be carried out in the future (accompanied with statistically powerful experimental designs) to better understand and verify the performance of fractal electrodes.

## Discussion and conclusion

This contribution presents proof-of-concept and preliminary results of using fractal electrodes to increase the efficiency of neural stimulation.

Recently, alternative electrode geometries have been introduced to improve the efficiency of neural excitation based on maximizing the electrode perimeter. The present work demonstrates another perspective for improved electrode geometries based on fractals. Fractal geometries have long been known for their particular topological properties such as non-integer capacity dimension, and the fact that they can theoretically have infinite perimeter while maintaining finite area. This makes fractal-shaped electrodes interesting candidates for use in neural stimulation, as they can increase the irregularity of current density profile on their surface, and consequently in the adjacent tissue. To investigate this argument, numerical simulations, and preliminary *in vivo* experiments were performed using modified Sierpinski square fractals. Numerical results predicted a substantial increase in the percentage of activated neurons and a reduction in the input power consumption by 22%. Preliminary results of median nerve stimulation using prototype electrodes were consistent with simulation results in one human subject. These promising observations warrant much more extensive *in vivo* testing.

The Sierpinski fractal has been chosen here because its geometry provides a natural alternative to square electrodes, which are already used in many neural stimulation applications such as transcutaneous electrical nerve stimulation (TENS), functional electric stimulation (FES) and as array elements in spinal cord stimulation and subdural cortical stimulation. However, other fractal shapes and complex geometries should be considered and tested. Other methods of increasing topological edginess, such as electrodes covered with a grid of insulating lines could also lead to useful increases in the activation function.

Future investigations should also focus on exploring the optimum configurations to enable activation *targeting* (maximizing activation along certain directions). Development of such capability could potentially provide significant advantage in targeting specific neural populations, allowing optimized therapeutic protocols.

**Figure 13:**
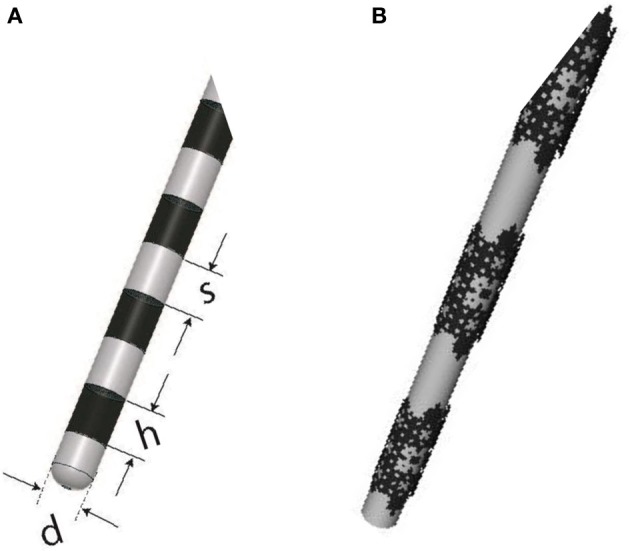
**(A)** DBS electrode contacts manufactured by Medtronic (ACTIVA 3387-Medtronic, Minneapolis, MN, USA) with *h* = 1.5 mm, *s* = 1.5 mm, and *d* = 1.27 mm, **(B)** A proposed alternative with solid conductive contacts replaced with a third order modified Sierpinski fractal.

Finally, it is important to note that fractal planar geometry can be extended to curved surfaces, and the resulting conductive stimulator should hypothetically work better than its conventional counterpart. For example, the cylindrical conductive electrodes used for DBS can be replaced by fractal electrodes, as illustrated in Figure 13, to enhance stimulation efficiency. Future experiments are planned to verify the performance of this new configuration.

### Conflict of interest statement

Two of the authors are inventors on intellectual property owned by Sunnybrook Research Institute and University of Toronto. If this intellectual property is licensed, then according to institutional revenue sharing policies, the authors may receive a share of fees and or royalty payments. The other authors declare that the research was conducted in the absence of any commercial or financial relationships that could be construed as a potential conflict of interest.

## Footnotes

^1^http://www.pcbfx.com/main_site/pages/start_here/overview.html
